# Sleeping sickness and its relationship with development and biodiversity conservation in the Luangwa Valley, Zambia

**DOI:** 10.1186/s13071-015-0827-0

**Published:** 2015-04-15

**Authors:** Neil E Anderson, Joseph Mubanga, Noreen Machila, Peter M Atkinson, Vupenyu Dzingirai, Susan C Welburn

**Affiliations:** The Royal (Dick) School of Veterinary Studies, Easter Bush Campus, The University of Edinburgh, Roslin, Edinburgh EH25 9RG UK; Division of Pathway Medicine and Centre for Infectious Diseases, School of Biomedical Sciences, College of Medicine and Veterinary Medicine, The University of Edinburgh, Chancellor’s Building, 49 Little France Crescent, Edinburgh, EH16 4SB UK; Department of Veterinary Services, Lusaka, Zambia; School of Veterinary Medicine, University of Zambia, Lusaka, Zambia; Geography and Environment, Highfield Campus, University of Southampton, Southampton, SO17 1BJ UK; Centre for Applied Social Sciences, Faculty of Social Sciences, University of Zimbabwe, Harare, Zimbabwe

**Keywords:** Trypanosomiasis, Tsetse, Wildlife, Biodiversity, Conservation, Sleeping sickness, Luangwa Valley, Zambia

## Abstract

The Luangwa Valley has a long historical association with Human African Trypanosomiasis (HAT) and is a recognised geographical focus of this disease. It is also internationally acclaimed for its high biodiversity and contains many valuable habitats. Local inhabitants of the valley have developed sustainable land use systems in co-existence with wildlife over centuries, based on non-livestock keeping practices largely due to the threat from African Animal Trypanosomiasis. Historical epidemics of human sleeping sickness have influenced how and where communities have settled and have had a profound impact on development in the Valley. Historical attempts to control trypanosomiasis have also had a negative impact on conservation of biodiversity.

Centralised control over wildlife utilisation has marginalised local communities from managing the wildlife resource. To some extent this has been reversed by the implementation of community based natural resource management programmes in the latter half of the 20^th^ century and the Luangwa Valley provides some of the earliest examples of such programmes. More recently, there has been significant uncontrolled migration of people into the mid-Luangwa Valley driven by pressure on resources in the eastern plateau region, encouragement from local chiefs and economic development in the tourist centre of Mfuwe. This has brought changing land-use patterns, most notably agricultural development through livestock keeping and cotton production. These changes threaten to alter the endemically stable patterns of HAT transmission and could have significant impacts on ecosystem health and ecosystem services.

In this paper we review the history of HAT in the context of conservation and development and consider the impacts current changes may have on this complex social-ecological system. We conclude that improved understanding is required to identify specific circumstances where win-win trade-offs can be achieved between the conservation of biodiversity and the reduction of disease in the human population.

## Introduction

### Biodiversity, conservation and disease

Worldwide economic growth and development over the last century has resulted in an unprecedented loss of biodiversity and a consequential reduction in ecosystem services [1]. Maintaining biodiversity and species richness is believed to reduce the risk of many diseases in people, animals and plants and to prevent disease emergence, thus providing an ecosystem service [1,2]. Whilst biodiversity has decreased during the last century, the rate of disease emergence has increased significantly [3]. A link between biodiversity losses and disease emergence has been shown most clearly for Lyme [2] and West Nile disease [4]. Although it is considered likely that biodiversity influences risk for many other vector-borne diseases in many other ecosystems, the effect is likely to depend on the specific local circumstances [5,6].

With a highly competent wildlife reservoir and a vector population that thrives in wild areas the ecology of trypanosomiasis has, arguably more than any other disease, been intertwined with the conservation of biodiversity. Historically there has often been a perceived conflict between the objectives of trypanosomiasis control and the objectives of conservation. Sir David Bruce was once famously quoted as recommending the “*early and complete blotting out”* of all wild animal hosts in areas inhabited by tsetse flies in order to control the disease [7]. In contrast, agricultural development has been regarded by many conservationists as the primary threat to the preservation of biodiversity, and the profound impact of tsetse-transmitted trypanosomiasis on land development has led to the fly being referred to by some as the ‘guardian of Africa’.

### Trypanosomiasis and the Luangwa Valley

The Luangwa Valley has a long history of disease due to human sleeping sickness and first achieved notoriety in the early 1900s when a new form of the disease was described [8]. Investigation showed a substantial proportion of the wildlife of the valley to be infected with trypanosomes [9]. Several surveys of the wildlife population have revealed an extensive reservoir of trypanosome infections, infective to human, wild and domestic animal populations. The vegetation of the Luangwa Valley supports high densities of *Glossina pallidipes*, *G. morsitans morsitans* and *G. brevipalpis* tsetse vectors of animal and human trypanosomes. The high level of trypanosome challenge has resulted in an almost complete absence of livestock in the valley. Human infection occurs through spillover from the wildlife reservoir and is characterised by a low level of endemic disease punctuated by occasional epidemics.

### Features of the Luangwa Valley

**Figure 1:**
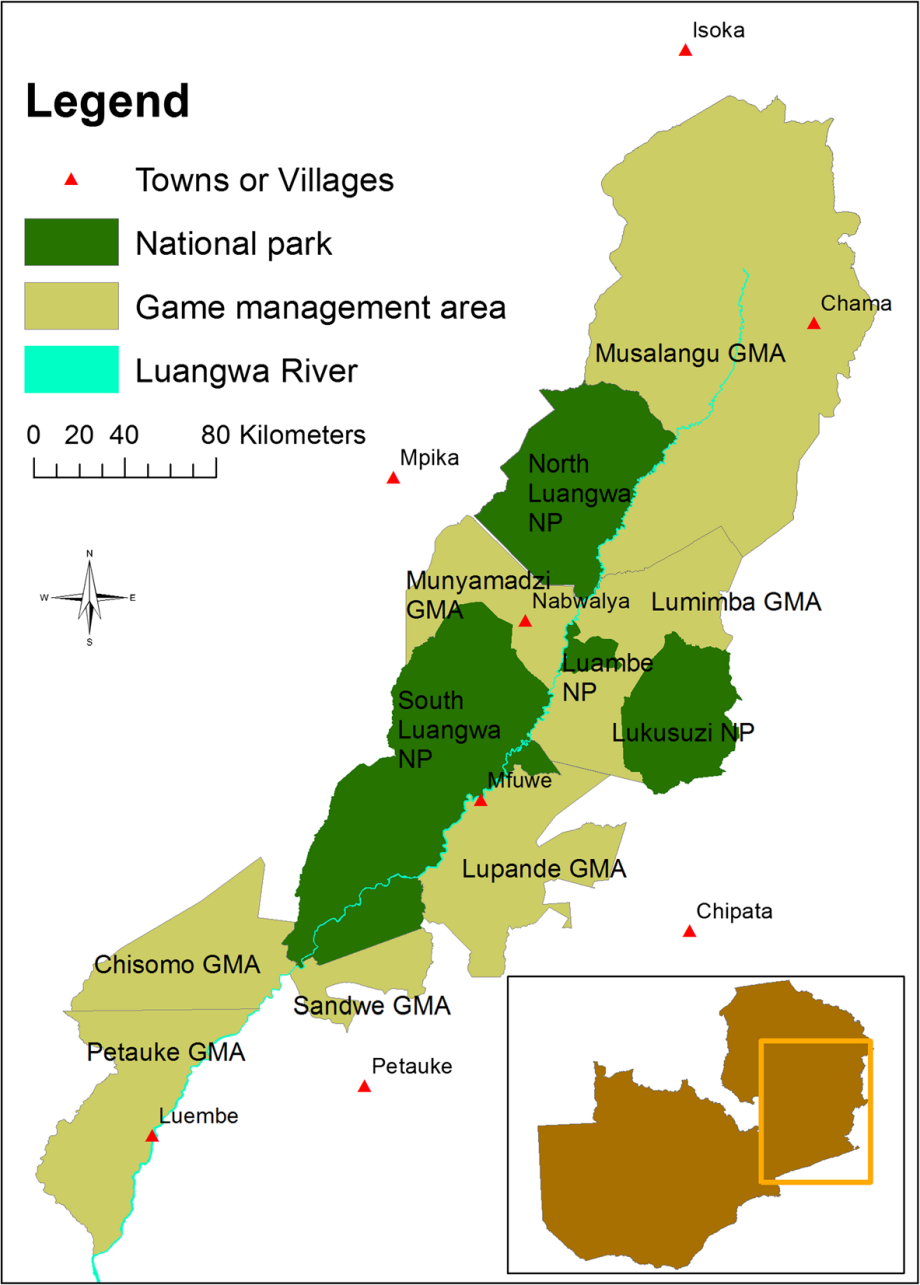
Map of the national parks and game management areas of the Luangwa Valley. Inset is an outline of the national boundary of Zambia showing the location of the Luangwa Valley.

The Luangwa Valley is a prominent geographical and geological feature in north eastern Zambia, covering some 45,000 km^2^ across the Muchinga, Eastern and Central Provinces of Zambia (Figure 1) [10]. The Luangwa River, flows for 700 km from its source in the Mafinga Hills of northern Zambia to its confluence with the Zambezi River in the south [11]. The valley climate is hot and dry with most of the rainfall occurring between November and March. Vegetation and climatic conditions on the valley floor are ideal for tsetse, but as altitude increases towards the escarpments bounding the valley the suitability for tsetse decreases. The valley hosts an internationally acclaimed diversity of wild fauna with the majority of the typical southern African savannah species represented. Additionally, it contains populations of several globally threatened wildlife species including black rhinoceros (*Diceros bicornis*), African painted dog (*Lycaon pictus*), African elephant (*Loxodonta africana*), Cookson’s wildebeest (*Connochaetes taurinus cooksoni*) and Thornicroft giraffe (*Giraffa camelopardalis thornicrofti*). The Luangwa Valley houses four national parks and has an international reputation for sport hunting on the game management areas (GMAs) surrounding the national parks.

### EcoHealth and threats to the endemic stability of trypanosomiasis in the Luangwa Valley

Patterns of land use in the Luangwa Valley have been relatively consistent over the past century, with only moderate increases in the human population density. The composition of the reservoir host community for trypanosomiasis has also remained stable since the first investigations of the sleeping sickness commission [12]. However, over the last decade there has been a significant departure from traditional patterns of land use in the mid-Luangwa Valley near Mfuwe. An influx of people from the over-populated plateau region of Eastern Province into Mambwe, Katete and Nyimba Districts has brought in new farming practices. Intensive cotton growing has become common, significant numbers of livestock have been introduced for traction [13] and activities such as logging and charcoal making have become institutionalised. Ecological changes associated with this diversion from traditional land use patterns will be contributing to reductions in biodiversity, ecosystem functioning and the ecosystem services provided. The rapid evolution of this novel human, domestic animal, wildlife and vector interface threatens the stable pattern of trypanosomiasis epidemiology observed over the last century [14]. Here, we review human sleeping sickness (Human African Trypanosomiasis, HAT) and nagana (African Animals Trypanosomiasis, AAT) in this historical focus of disease with particular reference to conservation of biodiversity. We explore the potential impacts the changes described above may have on this complex social-ecological system.

## Review

### Historical development in the Luangwa Valley

#### The pre-independence period

The early history of settlement in the Luangwa Valley has been reviewed in detail before [10,11]. Most of the present day inhabitants migrated from what is now the Democratic Republic of Congo from the early 16^th^ century onwards. Specialised techniques were developed to survive in what was a hostile environment. The high trypanosomiasis challenge made rearing livestock impossible and wildlife were hunted on a small scale as a protein source, supplemented by cultivation of sorghum, maize and millet. Hunting acquired cultural significance as well as being a key survival strategy [10]. The presence of vintage guns, particularly post-1800, suggested a growing hunting dynamic [15].

The Portuguese were recorded as exploring, trading and settling in the Luangwa Valley from the fifteenth century onwards [11]. A valuable trade network developed between the Bisa, Arabs and Portuguese for ivory, slaves and meat, with clear impacts on wildlife in the valley. These traders would in time create and finance hunting parties among the indigenous people, no doubt taking advantage of their detailed knowledge of the environment [15]. Wildlife populations were reduced, particularly outside tsetse belts where hunting by horse was less costly. From the late 1880s onwards hunting evolved from a subsistence or commercial activity into what has been described as ‘The Hunt’ – a symbol of ‘manliness’ and sportsmanship, but also of white dominance [15].

Towards the end of the nineteenth century the British gained political dominance and in 1889 the British South Africa Company (BSAC) was granted a Charter by the British Government. However, as dreams of mineral based wealth collapsed, early settlers and missionaries turned to farming and frequently utilised hunting to subsidise their activities and livelihoods. The new administration established regulations for the wildlife estate and imposed a ‘hut tax’ on valley inhabitants (payable in money, labour, grain or stock). Control reverted to the British government in 1924 and a policy of indirect rule was adopted based on indigenous tribal custom and government systems [11] although this may have just concealed despotism [16]. Native authorities were established with native courts and treasuries receiving a share of tax, license and court fees and central government grants. A change in administration formed the Federation of Rhodesia and Nyasaland in 1953, with present day Zambia becoming the Territory of Northern Rhodesia.

Settlement in the Valley now included development of colonial government structures and a regional administrative centre in Fort Jameson (now Chipata). Regulated sport hunting was initiated, but the rinderpest outbreaks of the 1890s decimated cloven-hoofed animal populations. The rinderpest outbreak resulted in the disappearance of the tsetse fly from large tracks of land [17,18] and it became possible to keep livestock in the Luangwa Valley for a limited period of time. The BSAC administrators maintained cattle and sheep at Nabwalya until 1905 [10] when the tsetse fly densities recovered [18]. There are records of livestock surviving until as late as 1916 in Chief Kakumbi’s lands [19]. Hunting became the preserve of early settlers with trade in wildlife products being highly lucrative. Cotton farming and mineral prospecting were also practiced [19].

#### The post-independence period

Zambia achieved independence in 1964 following a period of political upheaval in the Valley, particularly around Chipata (Fort Jameson). Economic development of the latter years of the protectorate continued into the 1960s and 1970s, resulting in migration of young men to the cities and the copper mines of northern Zambia. The density of local people inhabiting the valley was low during this period, ranging from 1.6/km^2^ to 3.3 km/^2^, despite having shown a steady increase from 1910 [20]. The poaching crisis of the 1970s and 1980s led to declines in wildlife populations and the local extinction of the black rhinoceros.

Recent decades have seen significant growth in human populations, particularly in the districts of Chipata, Katete and Chadiza which contain large urban populations (Table 1). A more modest increase has occurred in the districts within the Luangwa Valley itself. However, in recent years the mid-Luangwa Valley has seen significant changes in settlement patterns. Donor support for wildlife conservation and an increasing demand for tourism have created a substantial expansion of the tourist industry around Mfuwe. Coupled to this has been an influx of people from the eastern plateau into Mambwe and Katete district [13] seeking fertile soils for crops. Immigration, encouraged by Chief Kakumbi for micro-politics and patronage, has been accompanied by new farming techniques, environmental modification and an increase in the numbers of livestock kept by the new inhabitants.

**Table 1:** **Population density for the districts in and around the Luangwa Valley (1990, 2000 and 2010)**

**Name of district**	**Area (km** ^**2**^ **)**	**Population density (people/km** ^**2**^ **)**		
		**1990 census**	**2000 census (mean annual change, %)**	**2010 census (mean annual change, %)**
Chadiza	2574	25.9	32.6 (0.7)	41.7 (0.9)
Chama	17630	3.1	4.2 (0.1)	5.9 (0.2)
Chinsali	15395	5.8	8.4 (0.3)	9.5 (0.1)
Chipata	6693	39.0	54.9 (1.6)	68.1 (1.3)
Isoka	9225 (5091*)	8.9	10.8 (0.2)	14.2 (0.3)*
Katete	3989	36.1	47.4 (1.1)	61.1 (1.4)
Luangwa	3471	4.9	5.5 (0.1)	7.0 (0.2)
Lundazi	14058	12.8	16.8 (0.4)	23.0 (0.6)
Mambwe	5294	11.3	13.3 (0.2)	13.0 (0.0)**
Mpika	40935	3.0	3.6 (0.1)	5.0 (0.1)
Nyimba	10509	3.6	4.5 (0.1)	8.1 (0.4)

*Isoka District was divided into two districts in 2011 and the new district size was used for the 2010 census (new area shown in brackets). **The 2010 figures for Mambwe contain an anomaly as the population did not decrease between 2000 and 2010 [13].

## The history of human sleeping sickness in the Luangwa Valley

### Identification of *Trypanosoma brucei rhodesiense* and initiation of the sleeping sickness commission

The first documented case of Rhodesian sleeping sickness, as caused by *Trypanosoma brucei rhodesiense*, was published in 1910 [8]. A mineral prospector, W. Armstrong, became infected with sleeping sickness after visiting the Luangwa Valley in 1909. Laboratory investigations led to the discovery of a new form of human-infective trypanosome with differing virulence to *T. b. gambiense*, the only known cause of HAT at the time. Discovery of this new parasite, along with many other cases of trypanosomiasis in people visiting the Luangwa Valley, led to establishment of a sleeping sickness commission by the BSAC in 1911 [9]. The objective was to identify the vector and investigations were based at the abandoned government post at Nawalya (now Nabwalya, situated in Munyamadzi GMA) and on the western plateau at Ngoa.

The commission identified *G. morsitans* as the vector and showed a considerable percentage of wild animals were infected [9]. This was the first demonstration of trypanosomes in wild animals under natural conditions and antelope were considered to be the principal reservoir of the human trypanosome. Domestic livestock were scarce or non-existent in the Luangwa Valley at this time and cattle were recorded in only one village by the commission.

### Subsequent patterns of infection in the Luangwa Valley

Sleeping sickness in the Luangwa Valley has been characterised by low levels of endemic disease with occasional outbreaks and epidemics [21,22]. Epidemics of sleeping sickness resulted in a large area being declared a ‘Sleeping Sickness Area’ and closed to hunting by non-residents from 1912-1925 and from 1927-1934 [11]. Around this time Lane Poole stated that “*the presence of tsetse fly in great density and the occurrence of sleeping sickness in some localities deferred the traveller*” [19]. He described a severe epidemic of sleeping sickness affecting the people of Kakumbi in 1918 and numerous village relocations due to the presence of disease.

HAT has been most common in the north of the Valley in the Isoka and Chama Districts [21]. Buyst concluded that several factors could explain increased likelihood of disease in the north including; collision of an expanding fly belt with the human population, climatic stresses in the north of the valley and lack of abundant game animals as tsetse food source, and migratory wildlife movements. In an outbreak in Kasyasya village in Isoka District in 1982, some 11 cases were reported from a total population of 75 people [23]. Point prevalence of HAT in the northern Luangwa was estimated at 0.58% (using several parasitological methods and an indirect fluorescent antibody test (IFAT)) [22]. Disease incidence was estimated at 1%, with a 40% cumulative lifetime (40 years) risk of infection.

**Figure 2:**
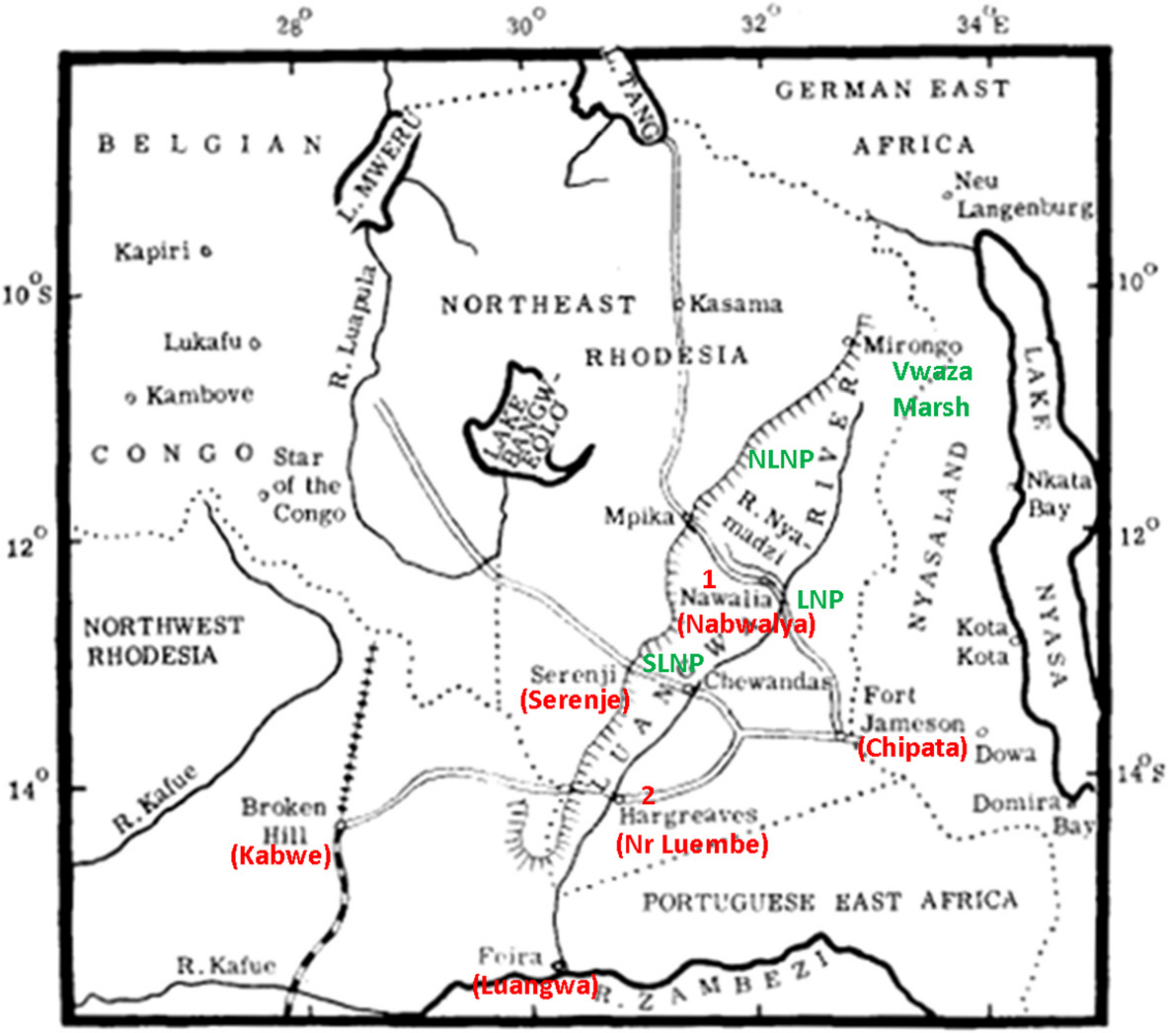
Map showing transit routes through the Luangwa Valley used by early traders and settlers. New place names are shown in red andapproximate locations of protected areas in green (NLNP – North Luangwa National Park; SLNP – South Luangwa National Park; LNP – Luambe National Park). 1:Site of sleeping sickness commission at Nawalia. 2: Site of abandoned river crossing at Hargreaves (near Luembe).Adapted from Willett [69], original taken with some additions from page 198 of Vol. 2 of The Sleeping Sickness Bulletin (1910).

HAT has also been reported in Petauke District, lower Luangwa Valley, and an early sleeping sickness focus was recorded at Hargreaves (Figure 2). Hargreaves was a Luangwa River crossing point which was abandoned due to its reputation as a source of sleeping sickness [24]. In 1971 an outbreak occurred resulting in 16 deaths out of 36 reported cases and reports emerged of ‘morbid mystique’ surrounding HAT [24], due to an extreme reluctance of people suffering from sleeping sickness to seek medical treatment. There was a belief that treatment would not result in a cure [25] and the occurrence of sleeping sickness in the Luangwa Valley has been associated with witchcraft and related accusation [26]. Examination of *Trypanozoon* taken from wild hosts between 1980 and 1983 using the blood incubation infectivity test (BIIT) [27] showed a large number of potentially human-infective parasites from animals in the upper Luangwa Valley around Kampumbu and Chibale (121) and fewer in the mid-Luangwa Valley around Mpika, Luchembe and Nabwalia (44). Very few potential human infective parasites were identified in Kakumbi, mid-Luangwa Valley and it was concluded that the risk in this area was small.

A controversial characteristic of sleeping sickness in the Luangwa Valley is the sub-acute nature of infection and existence of asymptomatic human carriers, postulated to be the result of long-term exposure and co-evolution [21,28,29]. A case-control study for sleeping sickness risk-factors in the northern Luangwa Valley found no increased risk in people born outside the valley and other studies have also questioned the existence of this sub-acute form of the disease [30-32].

More recently, official figures for sleeping sickness under-estimate the true disease incidence. Misdiagnosis and lack of diagnosis are common, in part due to the high incidence of retroviral disease [33].The distribution of HAT cases from 2000 to 2009 has been mapped as part of the HAT Atlas initiative, including the Luangwa Valley [34]. Within Zambia a total of 82 cases were recorded (including cases exported outside Zambia) with between 4 and 15 cases per year [34]. Cases have been reported in foreign tourists visiting the Luangwa Valley which could potentially impact negatively on tourism related activities [35]. A recent study estimated that approximately 416,000 people are at risk of contracting *T. b. rhodesiense* infection in Zambia although the risk is considered to be low in many areas [36].

### The wildlife reservoir for trypanosomiasis

There have been four major surveys of trypanosome infection in wildlife in the Luangwa Valley (Table 2). The first was conducted from 1911 to 1912 by Kinghorn *et al*. as part of the sleeping sickness commission described earlier [9]. A subsequent survey by Keymer in 1962 showed no significant change in the prevalence of trypanosome infections in wild ungulates during the intervening period [33]. Dillman and Townsend conducted a survey between 1971 and 1974, analysing 546 wild animal samples they found 79 infections, including nine of mixed species [37]. Using BIIT, human serum resistant parasites were isolated from two waterbuck and one warthog. They concluded that both species were natural reservoirs for human sleeping sickness [37].

**Table 2:** **Summary of trypanosomes detected in major surveys of wildlife in the Luangwa Valley**

**Species (n)**	**Survey reference**	***T. brucei***	***T. congolense***	***T. vivax***	**Total**	**Percentage positive**
African wild dog (5)	[12,37]	0	0	0	0	0.0
Baboon (20)	[37,70]	0	0	0	0	0.0
Bat (2)	[37]	0	0	0	0	0.0
Black rhinoceros (6)	[9,37]	0	0	0	0	0.0
Buffalo (88)	[12,37,71]	1	2	6	9	10.2
Bushbuck (66)	[9,12,37,71]	12	18	5	31**	47.0
Bushpig (4)	[9,71]	0	0	0	0	0.0
Cane rat (1)	[37]	0	0	0	0	0.0
Civit (6)	[37]	0	1	0	1	17.0
Crocodile (6)	[12,37]	0	0	0	0	0.0
Duiker (7)	[37]	0	2	1	3	42.9
Eland (4)	[12]	0	2	1	2*	50.0
Elephant (28)	[9,12,37]	0	2	0	2	7.1
Genet (6)	[37]	0	0	0	0	0.0
Giraffe (2)	[12,37]	1	0	0	1	50.0
Greater kudu (41)	[9,12,37,71]	0	19	7	18****	43.9
Grysbok (9)	[12,37]	0	0	0	0	0.0
Hare (10)	[37]	0	0	0	0	0.0
Hartebeest (11)	[9,12,71]	1	0	0	1	9.1
Hippopotamus (280)	[9,12,37]	5	0	1	6	2.1
Hyaena (14)	[12,37]	2	2	0	4	28.6
Impala (106)	[9,12,37,71]	4	2	0	7*	6.6
Jackal (1)	[37]	0	0	0	0	0.0
Leopard (16)	[12,37]	2	0	0	2	12.5
Lion (22)	[9,12,37]	5	7	0	12	54.5
Mongoose (2)	[37]	0	0	0	2	0.0
Puku (96)	[9,12,37,71]	2	2	2	6	6.3
Porcupine (1)	[37]	0	0	0	0	0.0
Roan (24)	[9,12,37]	0	3	0	3	12.5
Serval (2)	[37]	0	0	0	0	0.0
Vervet monkey (19)	[12,37]	0	0	0	0	0.0
Warthog (92)		5	9†	1	14*	15.2
Waterbuck (65)	[9,12,37,71]	7	6	26	43*****	66.2
Wild cat (1)	[37]	0	0	0	0	0.0
Wildebeest (20)	[9,12,37,71]	1	1	1	2*	10.0
Zebra (43)	[9,12,37,71]	0	0	0	0	0.0

*includes one mixed infection, **includes two mixed infections, ****includes four mixed infections, *****includes five mixed infections and †includes four *T. simiae* infections.

More recently, an extensive survey into the trypanosomiasis reservoirs in wildlife throughout the Luangwa Valley was undertaken, using novel molecular techniques for parasite identification [12]. Human-infective *T. b. rhodesiense* were confirmed in buffalo (*Syncerus caffer*) for the first time and were also identified in bushbuck. Bushbuck are considered the most significant reservoir host for *T. brucei* s.l. showing high levels of infection, along with waterbuck and the carnivores. Bovidae, and in particular greater kudu, were found to comprise the most important members of the host community for *T. congolense*. The prevalence of *T. vivax* was lower with waterbuck the most significant host identified. Other investigations in the Luangwa Valley have identified human serum resistant *T. b. rhodesiense* in warthog [38], zebra and impala [39]. *T. brucei* has been identified in four out of 75 hippo [40] and one lion [41]. The ecological factors associated with the epidemiology of wildlife trypanosomiasis have been reviewed in detail [42].

Over time, the wildlife reservoir in the Luangwa Valley appears relatively stable. Across all four surveys, the distribution of trypanosome infections is concentrated in three species of ungulate (bushbuck, waterbuck and kudu). This suggests that host susceptibility and vector ecology have not substantially altered in the last century. Although lion and other carnivores have frequently been identified as reservoir hosts, they are considered to contribute less epidemiologically since they can also become infected through ingestion of prey and their densities are relatively low being predators. As the general distribution of trypanosome infections within the wildlife community has been fairly well characterised already, further advancement of understanding is most likely to come from molecular investigations that examine host range and specificity of parasite infection (e.g. *T. godfreyi* recently identified in a mammalian host for the first time [43]), and that quantify the level of parasitaemia. The reliable identification of human infective *T. b. rhodesiense* in the wildlife reservoir has only recently become possible due to previous difficulties in diagnosing human infective parasites. Published identifications of *T. b. rhodesiense* in wild hosts using reliable methods are summarised in Table 3. The parasite has been detected in nine species, several of which are not preferred hosts for tsetse (giraffe, impala, waterbuck and zebra). It is possible that the lower level of challenge in these species makes them more susceptible to infection with the human infective parasite. Interestingly, waterbuck seem highly susceptible to infection with other trypanosome species as well [12]. In epidemiological terms, the bushbuck and warthog are considered the most important hosts due to their close ecological associations with tsetse. These species are sedentary and potentially contribute to the focal distribution of the disease [44,45]. The buffalo is also of interest as a host, despite being fed on less commonly by tsetse, as they are mobile species covering large distances which could disseminate infection.

**Table 3:** **Confirmed isolations of** ***T. b. rhodesiense*** **from wildlife using blood incubation and infectivity test (BIIT) or molecular tests (SRA-PCR)**

**Species**	**Technique**	**Reference**
Buffalo	SRA-PCR	[12]
Bushbuck	BIIT	[27]
Bushbuck	SRA-PCR	[12]
Duiker*	BIIT	[27]
Giraffe	BIIT	[27]
Impala	BIIT	[39]
Impala*	BIIT	[27]
Lion*	BIIT	[27]
Warthog	BIIT	[38]
Warthog	BIIT	[37]
Warthog	BIIT	[27]
Waterbuck	BIIT	[37]
Waterbuck*	BIIT	[27]
Zebra	BIIT	[39]

*Trypanosome isolates tested by Rickman et al [27] that were collected between 1971-1977 by the German Agency for Technical Cooperation, GTZ.

### Trypanosomiasis control and vector investigation

#### Localised control measures

The BSAC board were firm supporters of the principle of tsetse elimination in any area where land was required for people or their livestock [11]. The board considered that tsetse eradication should be achieved by elimination of wildlife hosts and they were profoundly influenced by the findings of the sleeping sickness commission. In line with colonial control policies of vilagisation aimed at maximising tax collection, local inhabitants were encouraged to live in larger villages to reduce exposure to tsetse challenge and many villages moved away from areas of high incidence. Game eradication became a major part of tsetse control policy in the Fort Jameson District [11,46]. The organisation of this typically involved safari hunting firms and commercial farmers with an interest in wildlife. This culling based form of control was popular due to the provision of game meat for the restless colonial labour and religious sceptics who came to church for that purpose.

Due to concern about the spread of tsetse from the valley, a scheme was initiated in 1944 to protect the commercial farmers on the eastern plateau by separating cattle from tsetse and a buffer zone was created, clearing woodland along a perimeter line and shooting any wild animals on the plateau side. By 1950, 9182 animals had been shot [11] and between 1956 and 1961, 2776 animals were killed (Table 4) [47]. A variety of species were shot, predominantly those that utilised habitat at a higher altitude further from the valley floor. Culled animals included the black rhinoceros which was to later become extinct in the Valley, and species such as roan and sable which are highly valuable in today’s market. Despite the relative abundance of wildlife at the time, these activities will have had a damaging effect on ecological health in the area and little positive effect on trypanosomiasis epidemiology in the wider Luangwa Valley due to the limited area covered. Other control policies adopted in Northern Rhodesia included discriminative bush-clearing and the spraying of insecticides (initially from the ground and later from the air) [46,48]. In the Luangwa Valley, control efforts were concentrated in the Fort Jameson District with little activity carried out further north away from commercial farming enterprises.

**Table 4:** **Summary of animals shot as part of tsetse control measures in Fort Jameson District (1956-1961)**

**Species**	**Year**						**Total**
	**1956**	**1957**	**1958**	**1959**	**1960**	**1961**	
Buffalo	51	46	57	58	27	28	267
Bushbuck	-	-	-	10	40	24	74
Bushpig	24	41	58	97	68	41	329
Duiker	-	-	-	40	133	210	383
Eland	20	31	26	36	12	8	133
Elephant	-	-	-	7	16	33	56
Grysbok	-	-	-	-	-	3	3
Hartebeest	8	21	25	17	-	-	71
Impala	1	2	-	5	-	-	8
Kudu	45	36	40	76	81	65	343
Oribi	-	-	-	-	2	4	6
Reedbuck	-	-	-	1	8	7	16
Rhinoceros	-	-	-	-	1	-	1
Roan	26	23	61	66	52	51	279
Sable	9	15	2	-	1	1	28
Warthog	57	100	139	186	97	112	691
Waterbuck	-	-	1	-	1	-	2
Zebra	13	27	20	26	-	-	86
**Total**	**254**	**342**	**429**	**625**	**539**	**587**	**2776**

Adapted from Clarke (1964) [47].

#### Regional control measures

Despite successes in controlling tsetse in many areas, the absence of effective barriers to re-invasion and the protracted war in the region between Zambia, Mozambique and Zimbabwe meant that many gains from earlier control measures were lost. As a result, several international collaborative efforts to eradicate tsetse were attempted. The first launched by the Food and Agriculture Organisation (FAO) in 1972, aimed to eradicate tsetse from Africa within ten years using insecticidal spraying [49].

Following the failure of this Africa wide programme to achieve its very ambitious aims, a regional approach to tsetse control in southern Africa was proposed. This proposal, referred to as the Regional Tsetse and Trypanosomiasis Control Programme (RTTCP), was funded by the European Union from 1984 and ran until the late 1990s. At inception, the objective of RTTCP was the eradication of tsetse from the entire common fly belt of southern Africa with a view to encouraging cropland farming, tourism and livestock production [50]. The regional approach was extractive and never incorporated indigenous means of either understanding or controlling tsetse. The focus changed to tsetse control in prioritised areas when it became clear that regional eradication was not achievable. By the end of the RTTCP it was widely considered ‘that the farmer must go it alone’ with respect to management of tsetse control.

**Figure 3:**
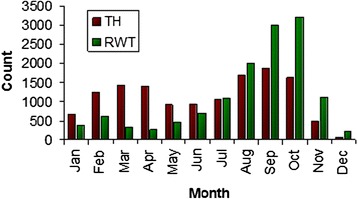
Mean monthly counts for *G. pallidipes* sampled in the SLNP using Epsilon traps. TH = thicket, RWT = Riverine woodland and thicket (source: RTTCP monthly reports for 1996 and 1997).

**Figure 4:**
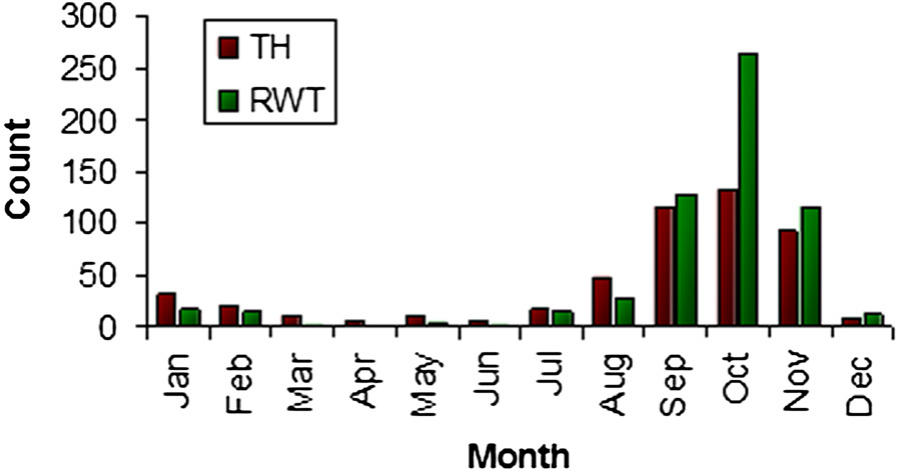
Mean monthly counts for *G. m. morsitans* sampled in the SLNP using Epsilon traps. Note: Scale is different from Figure 4 as counts for *G. m. morsitans* are much lower than for *G. pallidipes*(source: RTTCP monthly reports for 1996 and 1997).

South Luangwa national park (SLNP) was selected as a site for monitoring the effects of the RTTCP and as a consequence research into tsetse and trypanosomiasis was undertaken in the park and surrounding GMA. Considerable data was generated that included investigations into the distribution of trypanosome infections [51], age prevalence of cattle and tsetse populations [52,53], the dynamics of trypanosome infections [54] and the distribution of tsetse species using remotely sensed data and climate data [55,56]. Tsetse densities recorded during monitoring in the SLNP were high, illustrating the suitability of habitat in the Luangwa Valley. The graphs in Figure 3 and Figure 4 below illustrate monthly mean tsetse counts recorded in 1996 and 1997 for *G. pallidipes* and *G. m. morsitans* sampled using Epsilon traps. Clear seasonal fluctuations in counts are demonstrated in the two vegetation types sampled, particularly for *G. m. morsitans. Glossina brevipalpis* were also recorded during this period, but only in very small numbers because of the sampling method used and the occupation of a niche habitat close to rivers.

A new international collaborative approach to tsetse control was launched with the formation of the Programme Against African Trypanosomiasis (PAAT) in 1997. This programme sought to adopt a common strategy that would lead to a harmonised and standardised approach to trypanosomiasis control [49]. Demonstrating African governments continued support for tsetse eradication, the Organisation of African Unity (OAU) agreed to act collectively in a Pan-African Tsetse and Trypanosomiasis Eradication Campaign (PATTEC) “*in order to render Africa tsetse-free within the shortest possible time”* [49].

## Land use and protected area management in the Luangwa Valley

### Early land use strategies by local communities

High tsetse and trypanosomiasis challenge in the Luangwa Valley meant that agricultural practices involving livestock were not possible and the early settlers, the Bisa, successfully developed alternative strategies to manage the environment around them and to survive in a somewhat hostile environment [10]. These strategies to manage and utilise the local wildlife evolved and hunting became a main element of their culture. These strategies served to supplement dietary requirements (especially in times of drought), and to limit crop damage from wildlife. The advent of the trade in ivory, meat and hides by early explorers and traders lead to a change in the degree of utilisation of the wildlife, as well as the methods used. There were no restrictions on the numbers of wildlife killed by these early traders and the minimal annual commercial off-take of elephant from the Luangwa Valley in the mid-19^th^ century has been estimated to have been approximately 2200 animals [57].

### Creation of protected areas

The onset of the colonial era, however, brought the imposition of laws and regulations to control the utilisation of wildlife and sought ‘to protect the environment from the perceived threat from the local people’. These laws were implemented on a ‘fines and fences’ approach that can be traced back to the creation of the world’s first national park, Yellowstone, in 1872 [58]. Many of the inhabitants of the valley became marginalised and alienated by these new laws and many still have a negative view of conservation today [10].

The first set of regulations were adopted in 1900 [11] and the first game reserve was created in the Luangwa Valley in 1904 to protect giraffe populations. No development took place and it reverted back to its unprotected state in 1913. It was not until 1942 when control of the wildlife estate was passed over to The Department of Game and Tsetse Control that game reserves were once again created in the Valley. The southern and northern sections of the Luangwa Valley Game Reserve were gazetted and Controlled Hunting Areas were created. These measures were successful in conserving populations albeit through a top-down enforcement approach. After independence, the status of the game reserves was changed to that of national parks in 1972 and a system of GMAs replaced the Controlled Hunting Areas. In 2000, the semi-autonomous Zambian Wildlife Authority (ZAWA) was created to replace the National Parks and Wildlife Service.

### The current status of protected areas

Four national parks are recognised in the Luangwa Valley (Figure 1). The function of the national parks is to provide a breeding environment from which the wildlife population may ‘spill over’ into to the GMAs for consumptive purposes [59]. Non-consumptive tourism is very popular, especially in South Luangwa which is the highest revenue-earning park in Zambia (17853 international and 6076 domestic visitors in 2004 [60]). The Luangwa Valley also contains nine GMAs, many of which are classified as prime hunting areas. Together they cover a much larger area than the national parks and contain a wide diversity of wild flora and fauna. Consumptive utilisation of wildlife is allowed, but is regulated by the ZAWA. There are no restrictions on human settlement or agricultural practices in the GMAs. Theoretically, this policy could increase the number of people at risk of contracting sleeping sickness due to their proximity to intact tsetse habitat, but it must be remembered that people have lived in these areas for centuries and the creation of GMAs did not significantly alter populations. In conservation terms, GMAs have a conflicting function in that they are intended to provide buffers and wildlife corridors around and between national parks, but at the same time permit the utilisation of the wildlife contained within them for economic purposes.

### Community based natural resource management

The Luangwa Valley is notable for the degree of community engagement in natural resource management and provides some of the earliest examples of Community Based Natural Resource Management (CBNRM) programmes in Africa. From as early as 1941, local valley inhabitants received some benefit from wildlife under the colonial authority. In 1949, a ‘conducted hunting party scheme’ was initiated by Norman Carr with revenues going to the Native Authority [61] and the native authority reserve was created in Nsefu [11]. Despite these early progressive and inclusive policies, centralised control became strengthened towards the end of the colonial administration and during early independence. As a consequence of the poaching crisis of the late 1970s and early 1980s, two new approaches to CBNRM were trialled. The Luangwa Integrated Resource Development Project (LIRDP) and the Administrative Management Design for Game Management Areas (ADMADE) heralded a new era for community participation [62]. Although these programmes achieved mixed success [63], the CBNRM concept was strengthened under the Wildlife Act 1998 through the creation of Community Resource Boards and Village Action Groups managed by the newly created ZAWA. Such schemes are important in terms of sleeping sickness control, particularly after government policies of decentralisation and privatisation of veterinary services have left local communities to initiate control measures.

Another major development in the Luangwa Valley has been the move towards the creation of the Malawi/Zambia Transfrontier Conservation Area (TFCA) with the support of the International Union for Conservation of Nature (IUCN) [60]. If successful it is likely to improve biodiversity conservation and provide additional income for local communities through eco-tourism. It should also facilitate cross-border cooperation on disease control measures enabling more effective strategies for management of trypanosomiasis.

### Agricultural development

Agriculture, at subsistence or semi-commercial level, is the main occupation, revenue provider and source of food for the local valley inhabitants [64]. The Luangwa Valley is a challenging environment for agriculture with recurring droughts and widespread food insecurity [10]. Livestock keeping remains rare in the Luangwa Valley, due to the high trypanosomiasis challenge, problems with predation, a generally poor environment for domestic livestock, lack of veterinary support services and the prevalence of other diseases. A range of subsistence crops are grown including: maize, rice, beans, soya, groundnuts, sorghum, millet and cassava. Honey is harvested from wild bees and bee keeping is an increasingly popular livelihood strategy. Charcoal production is common in some parts of the valley, particularly Nyimba, Petauke and Chinsali districts which are situated towards centres of higher human density [42]. Many areas of the Luangwa Valley are highly suited for growing cotton [11] which is a popular cash crop for many communities. Restricted access to outside markets severely limits the benefits attainable in the Valley from agriculture.

### A new interface in the Mid-Luangwa Valley

It is unusual in 21^st^ century Africa for an area the size of the Luangwa Valley to have had such limited exposure to impacts from domestic livestock. The growth in human populations has been modest and land use activities have remained relatively consistent over the last century. Most districts in the Valley still lack livestock with the exception of poultry. However, the last decade has begun to see significant changes in the mid-Luangwa Valley with migration of people from the eastern plateau [13]. Concerns about lack of soil fertility and land for grazing, coupled with encouragement from some chiefs within the valley, have driven significant population migration into Mambwe and Katete Districts. This migration has been largely unregulated and unplanned by central government. The migrating people have introduced cattle, valued as a cultural resource and as draught power, into the ecosystem alongside new agricultural practices. Significant numbers of cattle, goats and pigs are now kept within Mambwe District (Table 5; data are provided for comparison from Mpika District, located at much higher altitude and largely outside the ecological limits of tsetse). Although human densities are lower in Mpika which will also impact on livestock density, it is remarkable how much higher the cattle densities are on the Valley floor in Mambwe. Expanding human development in the mid-Luangwa Valley has also been accompanied by more intensive cotton growing. Cotton production relies on sequential applications of pesticides (the synthetic pyrethroids deltamethrin and cyhalothrin, and neonicotinoids including acetamiprid are used in the Luangwa Valley). Tsetse flies are highly susceptible to pyrethroids and extensive insecticide application to cotton crops is likely to affect fly densities where cotton is grown.

**Table 5:** **Agricultural census figures for Mambwe District (source: Ministry of Agriculture)**

**Species**	**Mambwe district, 2006**		**Mpika district, 2007**	
	**Number**	**Density (km** ^**2**^ **)**	**Number**	**Density (km** ^**2**^ **)**
Pigs	4006	0.76	5631	0.14
Goats	8078	1.53	6495	0.16
Sheep	297	0.06	2788	0.07
Donkeys	15	0.00	12	0.00
Cattle	3704	0.70	4587	0.11
Dogs and Cats	3421	0.65	1633	0.04
Chickens	46863	8.85	23775	0.58
Ducks	1597	0.30	8933	0.22
Guinea Fowl	569	0.11	622	0.02
Pigeons	2558	0.48	692	0.02
Rabbits	54	0.01	531	0.01

Figures for Mpika District are included for comparison, representing higher ground areas with lower trypanosomiasis challenge.

Although small in relation to the Luangwa Valley itself, this new interface represents a significant diversion from traditional land use practices and has brought into question the widely held belief that livestock keeping is not possible. It is the first time large numbers of domestic livestock have been successfully kept since the rinderpest outbreak in the late 19^th^ century. It is an important development in terms of trypanosomiasis control as epidemics of AAT are more likely at new human/wildlife/livestock interfaces such as this [14].

## Ecosystem health and the transmission of trypanosomiasis

The health of ecosystems may be assessed in terms of their vigour, organisation and resilience [65]. Ecological changes that have a negative impact on any of these indicators are likely to reduce the ecosystem services available to the human population. Many of these services are vital for human health, including provision of clean water, regulation of floods and prevention of disease emergence amongst others. There is therefore an important question over what the changes in land use patterns in the Luangwa Valley will mean to the social ecology of the region and how it will impact on trypanosomiasis transmission.

Biodiversity has the potential to regulate the transmission of disease and, in particular, vector-borne disease [1,2]. For many vector-borne diseases, where biodiversity is maintained infection is less likely to be transmitted due to the abundance and diversity of wild hosts diluting the infection risk to humans [2]. Human risk is directly related to the likelihood of being bitten by an infected tsetse fly and this may be lower in the presence of abundant and diverse wild hosts, which represent a preferred meal for savannah species of tsetse (subgenus *Morsitans*). As certain host species appear to act as key members of the reservoir community for trypanosomiasis [12], the presence of a more diverse wild fauna is also likely to reduce the chances of a fly becoming infected by feeding on one of the key reservoir species.

However, the specific effects of a change in biodiversity on this complex disease system in the Luangwa Valley will depend on local ecological characteristics. Changes in the ecology, including loss of biodiversity coupled with human population migration, could potentially increase human-fly and livestock-fly contacts and, consequently, increase trypanosome transmission. This may be compounded by the presence of cattle in relatively high densities. Experience in East Africa has shown cattle to be highly competent reservoir hosts for HAT in the absence of wild hosts [66,67]. It is likely that trypanosomes in this part of the Valley, where there are few animal health control measures, will shift from a predominantly sylvatic cycle towards a cycle that is increasingly dependent on domestic animals. Intensive cotton production has the potential to reduce trypanosomiasis transmission, at least on a local scale, due to the toxic effects of pyrethroid insecticides on tsetse. Conversely, if cotton fields are abandoned due to poor returns, fallow land will make ideal tsetse habitat.

Fragmentation of habitat has important effects on populations and has been shown to reduce tsetse apparent density near the eastern plateau area towards Katete [68]. However, at lower altitudes on the Valley floor, intact patches of remaining undisturbed woodland vegetation communities are likely to continue supporting dense wildlife and vector populations. The juxtaposition of patches of new human settlement (coupled with livestock management and associated loss of *in situ* biodiversity) with these intact patches is of most importance. In particular, the necessity of humans and cattle to venture into these intact patches due to resource requirements (e.g., to water cattle) will ensure that humans come into contact with tsetse on a regular basis. Therefore, a positive correlation between biodiversity and disease is considered unlikely around the new interface in Mambwe District. Thus, while the juxtaposition hypothesis described above suggests a significant potential risk, the maintenance of high densities of wildlife populations in surrounding areas could represent at least a component of a potential win-win solution whereby sustainable ecosystem services are maintained while at the same time reducing the negative effects of ecosystems (human and cattle disease).

## Conclusions

Trypanosomiasis has impacted dramatically on both the development and administration of the Luangwa Valley. The ‘morbid mystique’ surrounding the human form of the disease and difficulties in its treatment have had profound effects on the societies of the Luangwa Valley and have had major influence over where people have settled [23]. The greatest impact of trypanosomiasis, however, has been its influence over land use systems that have been adopted in the Valley. The preclusion of livestock keeping from most areas of the Valley, except during a short period after the rinderpest epidemic, has dramatically affected the landscape and ecosystems that are present today. Indigenous people have developed a unique set of adaptations to manage the environment in the absence of livestock.

The recent developments in the mid-Luangwa Valley represent a significant departure from traditional land-use systems which will alter the patterns of disease transmission. Despite occurring in a relatively small area, the rate of change has been much greater than previously recorded which raises concerns about the potential (re-)emergence of communicable diseases under shifted socio-ecological system states. It also threatens to alter the health of the ecosystem which may impact negatively on ecosystem services and biodiversity conservation. While we have described what is happening in general terms and provided some valuable insights in this review, much uncertainty exists over the specific circumstances which lead to disease outbreaks. For places that are changing rapidly, such as the mid-Luangwa Valley, improved understanding is required to identify the specific states and thresholds that will favour an increase in disease transmission. Moreover, circumstances need to be identified where win-win trade-offs can be achieved in which ecosystem health and ecosystem services are sustained, and disease controlled, for long term human and animal wellbeing.

### Ethical statement

Authority for access to data and information cited in this review article was obtained from the Government of Zambia through the Director, Department of Veterinary and Livestock Development (DVLD), Ministry of Agriculture and Cooperatives.
